# Detection, Diagnosis, and Preventive Management of the Bacterial Plant Pathogen *Pseudomonas syringae*

**DOI:** 10.3390/plants12091765

**Published:** 2023-04-25

**Authors:** Piao Yang, Lijing Zhao, Yu Gary Gao, Ye Xia

**Affiliations:** 1 Department of Plant Pathology, College of Food, Agricultural, and Environmental Science, The Ohio State University, Columbus, OH 43210, USA; 2 OSU South Centers, The Ohio State University, 1864 Shyville Road, Piketon, OH 45661, USA; 3 Department of Extension, College of Food, Agricultural, and Environmental Sciences, The Ohio State University, Columbus, OH 43210, USA

**Keywords:** *Pseudomonas syringae*, pathogen detection, pathogen diagnosis, plant disease triangle, plant fitness tetrahedron, plant disease management hexagon

## Abstract

Plant diseases caused by the pathogen *Pseudomonas syringae* are serious problems for various plant species worldwide. Accurate detection and diagnosis of *P. syringae* infections are critical for the effective management of these plant diseases. In this review, we summarize the current methods for the detection and diagnosis of *P. syringae*, including traditional techniques such as culture isolation and microscopy, and relatively newer techniques such as PCR and ELISA. It should be noted that each method has its advantages and disadvantages, and the choice of each method depends on the specific requirements, resources of each laboratory, and field settings. We also discuss the future trends in this field, such as the need for more sensitive and specific methods to detect the pathogens at low concentrations and the methods that can be used to diagnose *P. syringae* infections that are co-existing with other pathogens. Modern technologies such as genomics and proteomics could lead to the development of new methods of highly accurate detection and diagnosis based on the analysis of genetic and protein markers of the pathogens. Furthermore, using machine learning algorithms to analyze large data sets could yield new insights into the biology of *P. syringae* and novel diagnostic strategies. This review could enhance our understanding of *P. syringae* and help foster the development of more effective management techniques of the diseases caused by related pathogens.

## 1. Introduction

Plant pathogen detection recognizes the presence of plant pathogens in a specific location or area. The process involves observing visible disease symptoms in plants, collecting plant samples for further laboratory analysis, or using remote sensing techniques to detect the presence of pathogens [1,2]. Plant pathogen detection aims to identify the presence of plant pathogens as early as possible so control measures can be implemented to alleviate their impacts on crop production [3]. On the other hand, plant pathogen diagnosis refers to identifying the specific cause of a plant disease. It involves the identification of specific disease-causing pathogens by biochemical, molecular, and other techniques [4]. Plant pathogen diagnosis aims to find the specific pathogen responsible for the specific disease so proper control measures can be implemented to limit the further spread of the pathogen and disease. Plant pathogen detection and diagnosis are critical to understanding and managing plant diseases. They are associated with applying multiple techniques and approaches to identify and understand the presence and cause of plant diseases [5,6].

Plant diseases are a significant constraint to crop production worldwide and exert particularly severe impacts in developing countries, where agricultural systems may be less resilient than in developed ones [7,8,9,10]. Although bacteria evolved billions of years ago [11], they had not been demonstrated to cause plant diseases until the late 19th century [12]. Bacterial plant diseases can reduce crop yields and debase the quality of harvested crops, thus leading to significant quality and economic losses for farmers and agricultural industries [13,14,15]. Studying bacterial plant pathogens helps identify the ways to detect, diagnose, prevent, and control those destructive plant diseases, such as using resistant crop varieties, applying chemicals or biological control agents, and implementing good agricultural practices [9,16]. By further understanding the biology and epidemiology of bacterial plant diseases, researchers can upgrade their strategies to reduce the impacts of these diseases on crop production and improve global food security. Alongside developing control measures, it is also essential to study bacterial plant diseases to understand the factors contributing to their emergence and spread [17]. These efforts can involve the identification of the genetic and environmental factors that influence plant disease development and the roles that different plant hosts, vectors, and reservoirs play in the transmission of bacterial plant pathogens.

Rapid detection and correct diagnosis of bacterial plant pathogens and diseases are increasingly essential for protecting global food security. By detecting and diagnosing these pathogens and diseases early, it is possible to implement control measures such as the application of chemicals or biological control agents or the implementation of other agricultural practices to reduce the impacts of these diseases on crop production [18]. Furthermore, bacterial plant pathogens can sometimes result in the contamination of human food with harmful pathogens [19]. By detecting and diagnosing these pathogens early, it is possible to implement the control measures to prevent food contamination and improve food safety [20]. Bacterial plant diseases can also sometimes lead to the extinction of plant species, particularly rare or endangered species [21]. By noticing these pathogens earlier, it is helpful to develop strategies to protect cultures or production directly.

There are challenges in detecting and diagnosing bacterial plant pathogens, such as *P. syringae* [22]. One challenge is the need for rapid and correct diagnosis of bacterial plant pathogens. However, traditional methods, such as biochemical or molecular techniques, can be time-consuming and may not provide rapid results [23]. Another challenge is adapting to the fluctuating environmental conditions [24]. Bacterial plant pathogens can be influenced by various factors, including temperature, humidity, soil conditions, etc., which can vary over time and space [20]. Therefore, it is difficult to accurately diagnose and control bacterial plant diseases as the effective control measures may vary, depending on the specific environmental conditions. Diagnosis and control require the development of flexible and adaptable diagnostic and control strategies tailored to the specific environmental conditions in which the diseases are occurring. There is also a need to account for the diversity of bacterial plant pathogens. A wide range of pathogens can cause bacterial plant diseases [25]. This diversity can make it difficult to accurately diagnose and control bacterial plant pathogens and diseases. The effective diagnostic and control strategies may vary depending on the pathogen involved.

## 2. *P. syringae* as a Bacterial Plant Pathogen

**Figure 1:**
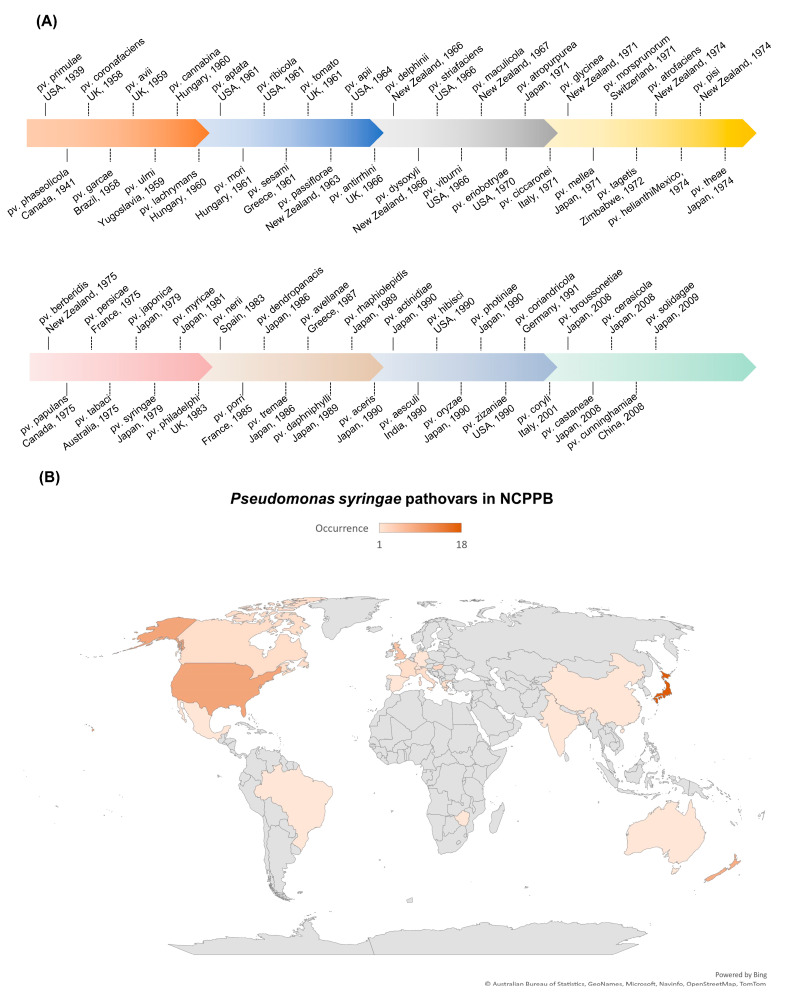
Occurrence of plant diseases caused by *P. syringae*. Data were retrieved from NCPPB (National Collection of Plant Pathogenic Bacteria, https://www.fera.co.uk/ncppb, accessed on 15 February 2023). (**A**) Landmark discoveries of pathovars of *P. syringae*. In the horizontal timeline, we only highlight those pathovars deposited to NCPPB. Due to space constraints, we were unable to cover all the significant discoveries that occurred along the timeline. (**B**) *P. syringae* pathovars deposited in NCPPB. The occurrence data were presented in a world map view. The color bar indicates the counts of different pathovars of *P. syringae* identified from the specific country until 2009. The gradient of the color (from lighter to darker orange) indicates the number/types of pathovars reported (from lower to higher level).

*P. syringae*, a Gram-negative, rod-shaped bacterium that can cause severe damage to many plant species, is a significant concern for plant health and crop production [26]. It is classified as a hemibiotrophic pathogen that initially feeds on living plant tissues and later causes the death of plant cells [27]. The *P. syringae* phylogenetic group includes more than 60 pathovars and 15 recognized bacterial species [28]. Each pathovar of *P. syringae* infects a distinct group of host plants and is known for its diverse host-specific interactions with the plants [29,30]. As early as 1939, the *P. syringae* pv. *primulae* was reported to cause necrotic leaf spots on primrose plants in the USA (Figure 1A) [31]. In 1961, the *P. syringae* pv. *tomato* was reported to cause necrotic leaf spots on tomato plants in the UK (Figure 1A) [32]. The *P. syringae* pv. *tomato* DC3000 is also pathogenic to Arabidopsis plants and has become a model pathogen for probing disease susceptibility and hormone signaling in plants [27]. Up to 2009, Japan witnessed the highest level of occurrence of plant diseases caused by *P. syringae*, followed by the USA (Figure 1B). Japan reported/deposited 18 different pathovars of *P. syringae* to the National Collection of Plant Pathogenic Bacteria (NCPPB), and the USA reported/deposited 9 different pathovars of *P. syringae* to NCPPB (Figure 1B), which increased our understanding of the occurrence/distribution of *P. syringae* on a world-scale view.

**Figure 2:**
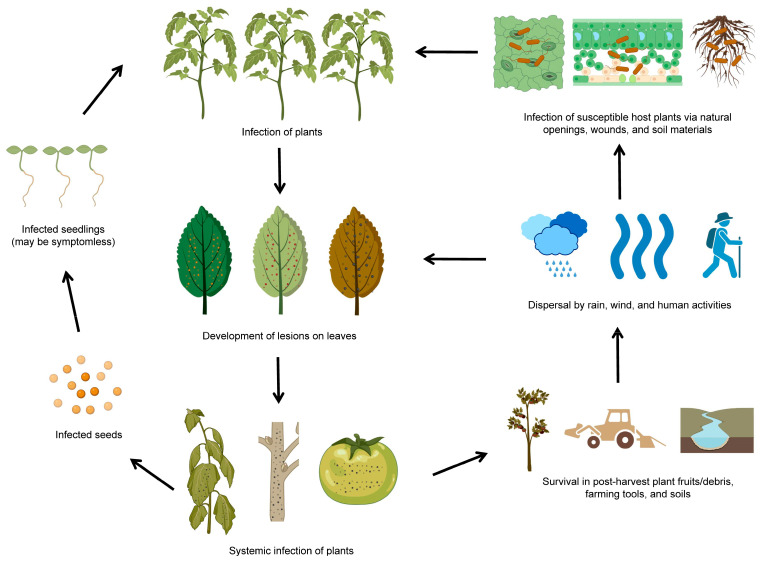
The life cycle of *P. syringae*. The diagram was adapted from [27] with some modifications and updates. The figure was created with BioRender.com, accessed on 15 February 2023.

The life cycle of *P. syringae* involves a range of different stages and modes of transmission [33]. *P. syringae* can be transmitted through seeds, water, vector insects, and infected plant debris. Once inside the plants, *P. syringae* can multiply and produce toxins that harm plant tissues. The infected plants can develop characteristic symptoms, such as lesions or discoloration on diseased leaves and necrosis spots on diseased fruits. *P. syringae* can survive in plant debris in the environment for extended periods and easily infect susceptible host plants through wounds or natural openings (Figure 2). It is worth noting that the life cycle of *P. syringae* can vary depending on the pathovar (strain) of the bacterium and the plant species it infects (Table 1). *P. syringae* is typically characterized by its ability to infect only specific areas of plants, such as foliar tissues and fruits. Some pathovars of *P. syringae* are more virulent or have a broader host range than others, affecting how the bacterium spreads and causes diseases [34].

*P. syringae* has been extensively studied since the early 1980s, and it is often used as a model for understanding various aspects of bacterial pathogenicity, including molecular mechanisms of plant-microbe interactions, microbial ecology, and epidemiology [27,30]. Genomic studies have revealed specific genomic characteristics that contribute to the virulence of *P. syringae*. Currently, it has been found that *P. syringae* deploys three vital strategies to harm plants: it can survive and adapt to the surface of plants, it can suppress the plant’s immune system at different stages of infection, and it can establish a water-filled space in the plant tissues, which provides it with the access to water and nutrients [30,92,93,94].

There are various techniques available for the detection and diagnosis of *P. syringae*. These techniques can be broadly classified into several categories: conventional (visual examination, microscopy, culture plate or phage typing), molecular (RPA, LAMP, NGS, FISH or PCR), serological (FCM, ELISA, IF or immunoStrip), biomarker-based (plant metabolite profiling, pathogen metabolite profiling, or microbiome analysis), vision-based (hyperspectral imaging or spectroscopic imaging) and AI (artificial intelligence). Different techniques have different advantages and limitations depending on the sample type, pathovar diversity, cost-effectiveness, etc. Conventional, molecular, and serological techniques are widely used nowadays for the detection and diagnosis of *P. syringae*.

## 3. Detection and Diagnosis of *P. syringae* with Conventional Methods

**Figure 3:**
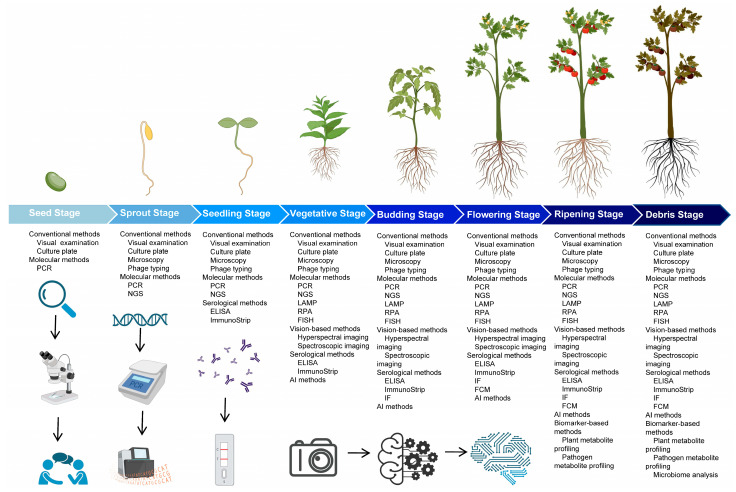
Methods in detection and diagnosis of *P. syringae* in the life history of a plant. The figure was created with BioRender.com, accessed on 15 February 2023.

Visual examination is commonly used to detect and diagnose plant diseases caused by diverse pathogens [95] (Figure 3). The advantages of visual examinations are as follows: (1) It is relatively quick and easy to perform, requiring no specialized equipment or training. (2) It allows the observer to examine the location and extent of the disease symptoms on the plants. (3) It can be performed in the field, allowing for real-time pathogen and disease assessment (Table 2). However, there are also some limitations to visual examination for plant pathogen detection and diagnosis: (1) It may not be sensitive enough to detect pathogens at the initial stages when symptoms may not be visible. (2) It is subjective, as different people may interpret the same symptoms differently. (3) It is affected by environmental conditions, such as lighting and weather, making it difficult to assess disease symptoms accurately. (4) It is not suitable for detecting diseases caused by pathogens not visible to the naked eye, such as viruses or bacteria that colonize inside the plant tissues (Table 2). In summary, one traditional method for identifying plant pathogens is through visual examination, but this approach is generally only practical after significant damage has already occurred. Moreover, treatments are often ineffective once visible damage has already taken place. Farmers need to be able to identify a pathogen before the symptom becomes apparent to prevent irreparable harm to crops [96,97].

Microscopy examination is a technique that uses a microscope to magnify and observe small objects, including plant tissues and cells (Figure 3) [17,18,19]. Under a microscope, *P. syringae* bacteria are rod-shaped and have a characteristic appearance. They are typically 1–2 μm in width and 2–5 μm in length. *P. syringae* cells are gram-negative, which means they have a thin cell wall and are stained pink by a Gram stain. The bacteria are motile and often have a single polar flagellum, which helps them move around [146,147]. In the context of plant pathogen detection and diagnosis of *P. syringae*, microscopy has several advantages: (1) High resolution: Microscopes can supply high-resolution images of plant tissues and cells, allowing for the detection of subtle features and abnormalities that may be indicative of disease. (2) Versatility: many diverse types of microscopes are available, each with unique capabilities, making microscopy a versatile tool for plant pathogen detection and diagnosis. (3) Accessibility: Microscopes are widely available and relatively inexpensive, making them accessible to researchers and practitioners in many different settings (Table 2). However, there are also some limitations to using microscopy for plant pathogen detection and diagnosis: (1) Sample preparation: Microscopy requires the preparation of thin sections or slides of plant tissues or cells, which can be time-consuming and may not be suitable for all types of samples. (2) Shallow depth of field: Microscopes have a limited depth of field, making it challenging to visualize objects that are not in focus. (3) Limited spatial resolution: Microscopes have a limited spatial resolution, making it difficult to distinguish between closely spaced objects or features. (4) Operator skill: The accuracy and usefulness of microscopy depend on the skill of the operator, who must be appropriately trained to properly prepare and observe samples [17,18,19] (Table 2).

Culture plates are a commonly used tool in *P. syringae* detection and diagnosis. They are flat dishes typically made of glass or plastic and are used to culture and preserve microbes, such as bacteria, fungi, and yeast (Figure 3) [103,104]. On a culture plate, *P. syringae* can form colonies that are typically smooth, circular, and slightly raised, with a glossy or opaque appearance. The color of the colonies can vary depending on the type of agar and the specific strain of *P. syringae*, but they are usually white, yellow, or cream-colored [148]. Culture plates have several advantages for plant pathogen detection and diagnosis: (1) Culture plates are relatively inexpensive and easy to use. (2) They supply a controlled environment for growing and keeping microbial cultures, which helps to ensure accurate and consistent results. (3) Culture plates allow for isolating individual microbial species, which is important for identifying and characterizing specific pathogens. (4) They can be used to identify the presence of a pathogen or co-existing pathogens in a sample by observing the growth of each pathogen on the individual culture plate (Table 2). However, culture plates also have some limitations: (1) Culture plates are not suitable for detecting all types of pathogens, as some pathogens may not grow well in culture. (2) They require a relatively long time to produce results, as microbial cultures need time to grow and develop. (3) Culture plates are prone to contamination, leading to false positives or negatives. (4) They do not supply information about the pathogenicity or virulence of a pathogen, which is essential for understanding its potential impacts on plants (Table 2). Overall, a culture plate is a useful tool for detecting and diagnosing plant pathogens. Still, culture plates should be combined with other techniques to provide a more complete picture of the pathogen and its impact on plants [103,104].

Phage typing is a method used to identify and characterize bacterial phages, which are viruses that infect bacteria. In plant pathogen detection and diagnosis, phage typing can be used to identify and differentiate bacterial pathogens that cause plant diseases (Figure 3) [105,106,107]. Advantages of phage typing include: (1) High specificity: Phage typing can be used to accurately distinguish between different bacterial strains, even those closely related. (2) Rapid results: Phage typing can supply results within a few days, making it a faster alternative than other methods, such as bacterial culture and biochemical testing. (3) Cost-effective: Phage typing is generally less expensive than other methods, such as DNA sequencing (Table 2). Limitations of phage typing include: (1) Limited to certain bacteria: Phage typing can be used only to identify bacteria susceptible to phage infection, which limits its applicability to a narrow range of bacterial pathogens. (2) Requirement for phage collection: Phage typing requires a collection of phages specific to the target bacteria, which may not be available for all bacterial species. (3) Limited resolution: Phage typing may not be able to distinguish between closely related bacterial strains, which can limit its accuracy. (4) Risk of contamination: Phage typing requires the handling of potentially infectious materials, which carries a risk of contamination (Table 2) [105,106,107].

## 4. Detection and Diagnosis of *P. syringae* by Molecular and Genetic Methods

Recombinase polymerase amplification (RPA) is a rapid, sensitive, and specific nucleic acid amplification technique that has been used in plant pathogen detection and diagnosis (Figure 3) [108,109,110]. RPA uses recombinase enzymes with accessory proteins to unwind and anneal primers to the target DNA or RNA [149]. Some advantages of RPA are as follows: (1) High sensitivity: RPA can detect low levels of target DNA or RNA, making it suitable for detecting pathogens at the preliminary stages of infection. (2) High specificity: RPA can distinguish between closely related pathogens, making it helpful in identifying specific pathogens in complex mixtures. (3) Rapid turnaround time: RPA can provide results within hours, making it useful for rapid diagnosis in plant disease outbreaks. (4) Easy to use: RPA does not require specialized equipment or complex protocols, making it accessible to many laboratories (Table 2). Some limitations of RPA are: (1) Limited multiplexing: RPA is not well suited for multiplexing (detecting multiple targets in a single assay), as it relies on specific primers and probes to detect target DNA or RNA. (2) Low throughput: RPA is not as efficient as the other amplification techniques, such as PCR, regarding the amount of DNA or RNA that can be amplified in a single reaction. (3) Poor stability: RPA reagents are prone to degradation, requiring frequent preparation and handling. (4) High cost: RPA reagents are more expensive than those used in other amplification techniques, such as PCR (Table 2). Overall, RPA has many potential applications in plant pathogen detection and diagnosis, but its limitations should be considered when choosing the most appropriate amplification technique for a given application [108,109,110].

Polymerase chain reaction (PCR) is a common laboratory technique widely used in plant pathogen detection and diagnosis as well as in many other fields of research and medicine (Figure 3) [104,116,117]. PCR amplifies small segments of genetic material using a polymerase enzyme and specific primers. For instance, PCR can detect *P. syringae* using primers based on different gene regions, such as the 16S–23S rDNA inter-transcribed spacer region [150]. There are several advantages to using PCR for plant pathogen detection and diagnosis: (1) Sensitivity: PCR can detect tiny amounts of DNA, making it extremely sensitive and able to detect even trace amounts of pathogens. (2) Specificity: PCR can specifically amplify a targeted region of DNA, making it highly specific and able to distinguish between different pathogens and genetic variations. (3) Speed: PCR can amplify DNA very quickly, making it possible to obtain results within a few hours or even minutes. (4) Versatility: PCR can be adapted to various applications and used to amplify DNA from various sources, including plant tissues, environmental samples, and clinical specimens (Table 2). There are also some limitations in using PCR for plant pathogen detection and diagnosis: (1) Cost: PCR requires specialized equipment and reagents, which can be expensive. (2) Complexity: PCR requires careful optimization and execution and can be challenging for those not experienced with the technique. (3) Limited sensitivity: In some cases, PCR may not be able to detect exceptionally low levels of pathogen DNA in a complex matrix. (4) False positives: PCR can produce false positive results if contaminants are present in the sample or if there are errors in the amplification process (Table 2). Overall, PCR is a powerful and widely used tool in plant pathogen detection and diagnosis, but it is important to consider both the advantages and limitations of the technique when designing and interpreting experiments [104,116,117].

Loop-mediated isothermal amplification (LAMP) is a rapid, sensitive, and specific method for amplifying DNA that has been widely used to detect and diagnose plant pathogens (Figure 3) [39,42]. For instance, LAMP can detect *P. syringae* using primers based on different genes, such as type III effector genes [151] or enolase genes [152]. Some advantages of LAMP for plant pathogen detection and diagnosis: (1) High sensitivity: LAMP can detect extremely low levels of target DNA, making it helpful in detecting pathogens at an early stage of infection. (2) Specificity: LAMP is specific to the target DNA, so it is unlikely to produce false positives or cross-react with other DNA sequences. (3) Rapid turnaround time: LAMP can amplify DNA in as little as 60 min, making it faster than the other amplification methods, such as PCR. (4) Simplicity: LAMP requires minimal equipment and can be performed at a single temperature, making it easy to use in various settings (Table 2). LAMP’s limitations for plant pathogen detection and diagnosis: (1) Limited multiplexing capabilities: LAMP is typically limited to amplifying a single target DNA sequence at a time, so it is not suitable for analyzing multiple targets simultaneously. (2) Poor performance with complex DNA templates: LAMP can be less efficient at amplifying DNA from complex samples, such as those holding multiple pathogens or contaminants. (3) Inability to detect DNA deletions or insertions: LAMP does not detect changes in the DNA sequence, such as deletions or insertions, so it may not be suitable for certain types of genetic analysis. (4) Limited commercial availability: LAMP kits and reagents are not as widely available as those for the other amplification methods, such as PCR (Table 2) [108,111].

Next-generation sequencing (NGS) is a high-throughput DNA/RNA sequencing technology that allows scientists to sequence copious amounts of DNA in a single experiment rapidly and accurately. NGS has revolutionized the field of genomics by providing researchers with the ability to analyze entire genomes, exomes (the part of the genome that codes for proteins), and transcriptomes (the set of all RNA molecules in one cell or a population of cells) at an unprecedented level of detail. NGS is used in various applications, including genomic sequencing, gene expression analysis, metagenomic analysis, genetic variation analysis, gene function analysis, etc. NGS is an important tool in many areas of research, including genetics, genomics, and medicine, and has contributed to numerous scientific discoveries and innovations (Figure 3) [112,113]. The analysis of the plant-associated microbiome compositions and functions through the NGS approach, such as metagenomic analysis, refers to the collective genetic/genomic information of the microorganisms that live in and on a plant, which has the potential to be a powerful tool in the detection and diagnosis of *P. syringae* or those pathogens co-existing with *P. syringae* (Figure 3) [128,134]. For instance, NGS can detect *P. syringae* by sequencing its whole genome or specific regions, such as the 16S rRNA gene or multilocus sequence typing (MLST) loci. There are several advantages of using next-generation sequencing (NGS) in plant pathogen detection and diagnosis: (1) High throughput: NGS allows researchers to analyze large amounts of DNA data in a single experiment, which is useful for detecting and diagnosing plant pathogens that affect many plants, such as *P. syringae* and the other pathogens co-existing with *P. syringae*. (2) High accuracy: NGS generates high-quality, reliable, accurate data, which is essential for accurately detecting and diagnosing plant pathogens. (3) Multiplexing: NGS allows researchers to analyze multiple samples simultaneously, useful for studying plant pathogens and diseases in different environments or at various stages of development (Table 2). There are also some limitations to using NGS in plant pathogen detection and diagnosis: (1) Cost: NGS can be expensive, especially when substantial amounts of data or multiple samples are analyzed. (2) Complexity: NGS requires specialized equipment and expertise, which can be a barrier to using this technology in some research settings. (3) Data analysis: NGS generates enormous amounts of data that can be difficult to analyze and interpret, especially for researchers with limited bioinformatics experience. (4) Sensitivity: NGS may not be sensitive enough to detect low levels of pathogen-associated DNA, which can limit its usefulness in some cases (Table 2). Overall, NGS is a powerful tool for detecting and diagnosing bacterial pathogens, such as *P. syringae*, but it is essential to take both the advantages and limitations into consideration when designing and implementing studies [112,113].

Fluorescence in Situ Hybridization (FISH) is a molecular biology technique that uses fluorescent probes to detect and locate specific DNA or RNA sequences in cells or tissues (Figure 3). FISH can detect DNA and RNA by hybridizing complementary probes that are labeled with different fluorophores and visualize the location and distribution of target sequences by using a fluorescence microscope [153]. FISH has several advantages for detecting and diagnosing *P. syringae* [114,115]: (1) High sensitivity: FISH can detect single copies of DNA or RNA sequences in cells, making it more sensitive than the other techniques such as microscopy. (2) High specificity: FISH can detect specific DNA or RNA sequences with high accuracy, making it more specific than techniques such as PCR, that amplify all DNA or RNA sequences in a sample. (3) Rapid: FISH can be completed relatively quickly compared to the other techniques, such as DNA sequencing. (4) Easy to visualize: FISH uses fluorescent probes, which allow researchers to easily visualize the presence of specific DNA or RNA sequences under a fluorescence microscope (Table 2). Limitations: (1) Expensive: FISH requires specialized equipment and reagents, making it more expensive than the other techniques. (2) Time-consuming: FISH requires careful preparation of samples, which can be time-consuming. (3) Requires trained personnel: FISH requires specialized training to perform and interpret, which can be a limitation in some settings. (4) Limited to specific sequences: FISH can only detect specific sequences of DNA or RNA designed as probes, so it is limited to the available sequences (Table 2). Overall, FISH is a powerful technique for plant pathogen detection and diagnosis, but it has certain limitations that should be considered when selecting the appropriate method for a given study [114,115].

## 5. Detection and Diagnosis of *P. syringae* with Serological Methods

Flow cytometry is a technique that uses lasers and specialized detectors to measure the physical and chemical characteristics of cells or particles suspended in a fluid. Flow cytometry can detect cells or particles by labeling them with fluorescent markers that bind to specific molecules, such as DNA, RNA, proteins, or antibodies. Flow cytometry is often used in plant pathogen detection and diagnosis as it allows researchers to rapidly and accurately quantify and analyze large numbers of cells or particles (Figure 3) [118,119,120]. For instance, flow cytometry can detect *P. syringae* by staining them with fluorescent antibodies that recognize specific antigens on their surface [154]. There are several advantages to using flow cytometry in *P. syringae* detection and diagnosis: (1) High throughput: Flow cytometry can analyze thousands of cells or particles per second, making it a highly efficient and rapid method for analyzing large samples. (2) Multiple parameters: Flow cytometry can simultaneously measure multiple physical and chemical characteristics of cells or particles, allowing researchers to obtain a comprehensive and detailed sample analysis. (3) Sensitivity: Flow cytometry is highly sensitive and can detect small changes in cell or particle characteristics, making it useful for detecting pathogens at the early stages of diseases or identifying subtle changes in cell function. (4) Accuracy: Flow cytometry is an accurate method with low error rates and high reproducibility for analyzing cells or particles (Table 2). There are also some limitations to using flow cytometry in plant pathogen detection and diagnosis: (1) Complexity: Flow cytometry requires specialized equipment and trained personnel to run it, making it a more complex and expensive method than the other techniques. (2) Sample preparation: Flow cytometry requires samples to be suspended in a fluid, which can be a time-consuming and labor-intensive process for plant samples. (3) Limited cell types: Flow cytometry is the most effective approach for analyzing cells or particles suspended in a fluid and may not be suitable for analyzing solid tissue samples or cells with thick walls. (4) Interference: Flow cytometry can be influenced by factors such as particles or contaminants in the samples, which can affect the accuracy of the results (Table 2) [118,119,120].

Enzyme-linked immunosorbent assays (ELISAs) are a type of immunoassay widely used in plant pathogen detection and diagnosis (Figure 3) [121,122,123]. ELISAs can detect molecules by binding them to a solid surface, such as a microplate well, and adding an enzyme-labeled antibody that recognizes the target molecule [155]. For instance, ELISAs can detect *P. syringae* using antibodies that recognize specific antigens on the surface, such as the lipopolysaccharides or flagellins [156]. Some advantages of ELISAs for plant pathogen detection and diagnosis are as follows: (1) Sensitivity: ELISAs are highly sensitive and can detect very low levels of specific antibodies or antigens in a sample. This makes them useful for detecting plant pathogens in the preliminary stages when the levels of specific markers may be low. (2) Specificity: ELISAs are specific for the antibodies or antigens they are designed to detect, allowing for the accurate identification of specific plant pathogens. (3) Speed: ELISAs can be completed relatively quickly, often within a few hours, making them a convenient and efficient tool for plant pathogen diagnosis. (4) Versatility: ELISAs can be modified to detect a wide range of plant pathogens, making them versatile for plant pathogen and disease diagnosis (Table 2). Some limitations of ELISAs for plant pathogen detection and diagnosis: (1) Complexity: ELISAs require a series of steps and specialized equipment, making them more complex and time-consuming compared to other diagnostic techniques. (2) Cost: ELISAs need specialized reagents and equipment, which can make them more expensive than the other diagnostic techniques. (3) Limited scope: ELISAs are limited to detecting specific antibodies or antigens, which may not be present in all plant pathogens. This can limit their usefulness for the diagnosis of some plant pathogens. (4) False positives: ELISAs can sometimes produce false positive results, which may lead to incorrect diagnoses. This can be a particular concern when ELISAs are used to diagnose rare or unusual plant pathogens (Table 2) [121,122,123].

Immunofluorescence is a technique that uses antibodies labeled with fluorescent dyes to detect and visualize specific proteins or other molecules in a sample. Immunofluorescence can detect antigens using a microscope that excites the fluorescent molecules and captures their emitted light [157]. In plant pathogen detection and diagnosis, immunofluorescence can be used to identify the presence of specific pathogen-associated molecular patterns (PAMPs) or effector proteins, which are secreted by pathogens and contribute to disease development (Figure 3) [102,124,125]. Some advantages of immunofluorescence for plant pathogen detection and diagnosis are the following: (1) High sensitivity: Immunofluorescence is highly sensitive and can detect low levels of specific molecules in a sample. (2) High specificity: Immunofluorescence is specific to the target molecule and can differentiate between closely related molecules. (3) Multiplexing: Immunofluorescence can detect multiple target molecules in the same sample, allowing for the analysis of multiple aspects of the disease process at once. (4) Ease of use: Immunofluorescence is a relatively simple and straightforward technique that can be performed in most research laboratories (Table 2). However, there are also some limitations to using immunofluorescence for plant pathogen detection and diagnosis: (1) Requirement for specific antibodies: Immunofluorescence needs specific antibodies that recognize the target molecule, which can be challenging to obtain or produce. (2) Limited to surface-exposed molecules: Immunofluorescence can only detect molecules that are accessible on the surface of cells or tissues and may not be able to detect molecules that are hidden inside cells or tissues. (3) Interference from the other molecules: Immunofluorescence signals can be masked or confused by the other molecules in the sample that cross-react with the antibodies. (4) Need for specialized equipment: Immunofluorescence requires the use of specialized equipment, such as fluorescence microscopes, which can be expensive and may not be available in all laboratories (Table 2) [102,124,125].

ImmunoStrip is a rapid diagnostic test that uses lateral flow technology to detect specific plant pathogens in a sample. ImmunoStrip can detect antigens using a sample pad that absorbs the liquid sample and transfers it to a test zone where the antigen binds to a capture antibody coated on a membrane [158]. ImmunoStrip tests are designed to be simple, fast, and easy to use, and they can provide results within a few hours (Figure 3) [122,126]. One of the main advantages of ImmunoStrip is that it is a non-destructive method of testing, which means that it does not require the significant destruction of plant tissues. This makes ImmunoStrip an attractive option for *P. syringae* detection and diagnosis, particularly in cases where plant tissue is limited or valuable (Table 2). However, there are also some limitations to ImmunoStrip. One limitation is that ImmunoStrip is a qualitative test, which means it can only detect the presence or absence of a specific pathogen rather than quantifying the amount of pathogen present in the sample. Another limitation is that ImmunoStrip may only be suitable for detecting some types of plant pathogens as it depends on the availability of specific antibodies that can bind to the pathogen of interest. In addition, ImmunoStrip tests can be affected by the presence of other substances in the sample, such as contaminants or plant compounds, which may interfere with the test results. Finally, ImmunoStrip tests may not be as sensitive as some other diagnostic methods, such as PCR (polymerase chain reaction), which can detect trace amounts of the pathogen in a sample (Table 2) [122,126].

## 6. Detection and Diagnosis of *P. syringae* with Biomarker-Based Methods

A biomarker is a measurable indicator of a biological state or condition that helps distinguish a diseased plant from a healthy one [159]. Biomarkers can be used to develop accurate and sensitive methods in the detection and diagnosis of plant pathogens [160]. For instance, biomarker-based methods can detect *P. syringae* by using biomarkers that are specific to the infection or metabolism, such as lipopolysaccharides (LPS), flagellins (FLG), quorum sensing signals (QSS), coronatine (COR), or salicylic acid (SA) [161]. Plant metabolite profiling (metabolomics) is a technique that involves the analysis of the metabolites present in plant tissues, which involves using nuclear magnetic resonance (NMR) or mass-spectrometry (MS) coupled with liquid chromatography (LC) or gas chromatography (GC) [162,163,164]. This technique uses the metabolites as the biomarkers to detect and diagnose plant pathogens, including *P. syringae* (or those pathogens co-existing with *P. syringae*), by identifying changes in the levels of specific metabolites that are associated with disease development (Figure 3) [127,128,129]. There are several advantages to using plant metabolite profiling in plant pathogen detection and diagnosis: (1) High sensitivity: Plant metabolite profiling can detect slight changes in the levels of specific metabolites, which may be indicative of early stages of disease development. (2) Multiplexing capability: Plant metabolite profiling can simultaneously measure the levels of multiple metabolites, providing a comprehensive view of the plant’s metabolic status. (3) High-throughput: Plant metabolite profiling can be performed on large numbers of samples in a brief period, making it a high-throughput technique (Table 2). However, there are several limitations to using plant metabolite profiling in plant pathogen detection and diagnosis: (1) Complexity: Plant metabolite profiles can be complex with hundreds or thousands of different metabolites present. This complexity can make it difficult to interpret the results of plant metabolite profiling studies. (2) False positives: Plant metabolite profiling can generate false positive results, as changes in the levels of certain metabolites may not always be directly related to disease development. (3) Limited specificity: Plant metabolite profiling may not be specific for a particular pathogen, as changes in metabolite levels may be caused by other factors such as environmental conditions or genetic variations. (4) Expensive: Plant metabolite profiling can be expensive due to the specialized equipment and expertise required (Table 2). Overall, plant metabolite profiling is a powerful tool for plant pathogen detection and diagnosis, but its effectiveness is limited by the complexity of plant metabolism and the potential for false positive results [127,128,129].

In addition to plant metabolite profiling, we can also carry out plant pathogen metabolite profiling. There are several advantages of using pathogen metabolite profiling in *P. syringae* detection and diagnosis (Figure 3) [130,131,132,133]: (1) Specificity: Metabolite profiling is specific to the pathogen of interest, which makes it a highly reliable method for detecting and diagnosing plant pathogens. (2) Early detection: Metabolite profiling can detect plant pathogens at an early stage, which is useful for implementing timely control measures. (3) Multiplexing: Metabolite profiling allows researchers to analyze multiple samples simultaneously, which is useful for studying plant pathogens in different environments or at various stages of development (Table 2). There are also some limitations to using pathogen metabolite profiling in plant pathogen detection and diagnosis: (1) Cost: Metabolite profiling can be expensive, especially when analyzing large amounts of data or multiple samples. (2) Complexity: Metabolite profiling requires specialized equipment and expertise, which can be a barrier to using this technology in some research settings. (3) Data analysis: Metabolite profiling generates substantial amounts of data that can be difficult to analyze and interpret, especially for researchers with limited bioinformatics experience. (4) Sensitivity: Metabolite profiling may not be sensitive enough to detect low levels of disease-associated metabolites, limiting its usefulness in some cases (Table 2). Overall, pathogen metabolite profiling is a powerful tool for detecting and diagnosing plant pathogens, but it is important to consider both the advantages and limitations of this technology when designing and implementing studies [130,131,132,133].

We can also carry out the plant-associated microbiome metabolite analysis. Some of the potential advantages of microbiome metabolite analysis in this context: (1) Early detection: Changes in the microbiome can occur before visible symptoms of the disease appear, allowing for the detection of pathogens in their early stages. (2) Broad coverage: Microbiome analysis can provide information about a wide range of microorganisms that may be present in or on a plant, including bacteria, fungi, and viruses. (3) Potential for early warning: Changes in the microbiome can serve as an early warning of impending pathogens and diseases, allowing for the implementation of preventive measures (Table 2). However, there are also some limitations to microbiome analysis in plant pathogen detection and diagnosis: (1) Complexity: The microbiome is complex and dynamic and can be influenced by various factors such as the environment, host genetics, and other microorganisms. (2) Limited understanding: Our understanding of the roles of individual microorganisms in the microbiome is still limited, and more research is needed to fully understand the relationships between different microorganisms and the hosts. (3) Technical challenges: Analyzing the microbiome requires specialized equipment and expertise, and the data generated can be complex and challenging to interpret. (4) Limited predictive power: While changes in the microbiome can be correlated with plant pathogens and diseases, they may not necessarily be causally linked. Further research is needed to understand the specific mechanisms by which the microbiome and related metabolites may influence plant health (Table 2) [128,134].

## 7. Detection and Diagnosis of *P. syringae* with Vision-Based Methods

Hyperspectral imaging is a technique that involves the acquisition and analysis of spectral data from a scene or object using a spectrometer or the other imaging instrument (Figure 3) [135,136,137,138]. In plant pathogen detection and diagnosis, hyperspectral imaging has several advantages, including the following: (1) Rapid: Hyperspectral imaging can provide rapid results, allowing researchers to quickly identify diseased plants and take appropriate actions. (2) High-throughput: Hyperspectral imaging allows researchers to analyze large numbers of plants quickly and efficiently, making it a high-throughput method for pathogen detection and diagnosis. (3) High spatial and spectral resolution: Hyperspectral imaging provides high spatial and spectral resolution, allowing researchers to analyze small features and subtle changes in plant tissues (Table 2). Despite these advantages, hyperspectral imaging has several limitations in plant pathogen detection and diagnosis: (1) Complex data analysis: The large amount of data generated by hyperspectral imaging can be difficult to analyze, requiring specialized software and expertise. (2) Cost: Hyperspectral imaging equipment can be expensive, limiting its use in some research settings. (3) Limited penetration depth: Hyperspectral imaging is limited in its ability to penetrate deep into plant tissues, making it less effective for detecting pathogens that affect internal plant organs. (4) Environmental factors: Hyperspectral imaging can be affected by environmental factors such as light intensity and atmospheric conditions, which can affect the accuracy of the results (Table 2). Overall, hyperspectral imaging is a powerful tool for plant pathogen detection and diagnosis, but it is important to consider its advantages and limitations in the context of specific research goals and objectives [135,136,137,138].

Spectroscopic imaging is a technique that involves the use of spectroscopy to obtain images of samples based on their spectral properties. Spectroscopic imaging has several advantages and limitations in the detection and diagnosis of *P. syringae* (Figure 3) [139,140]. Advantages of spectroscopic imaging in plant pathogen detection and diagnosis: (1) High spatial resolution: Spectroscopic imaging can provide high spatial resolution images, allowing for the detection of small changes in plant tissues that may not be visible using other techniques. (2) High sensitivity: Spectroscopic imaging is highly sensitive and can detect small changes in the spectral properties of plant tissues that may be indicative of disease. (3) Rapid analysis: Spectroscopic imaging can provide rapid analysis of plant samples, making it useful for large-scale pathogen and disease screening and diagnosis (Table 2). Limitations of spectroscopic imaging in plant pathogen detection and diagnosis: (1) Limited depth penetration: Spectroscopic imaging is limited by the depth of penetration of the spectroscopic signal, which can be affected by the size and density of the plant tissues. (2) Complex sample preparation: Spectroscopic imaging may require complex sample preparation techniques, such as slicing or sectioning the sample, which can be time-consuming and labor-intensive. (3) Need for calibration: Spectroscopic imaging relies on calibrated standards, which may be difficult to obtain for certain plant pathogens. (4) Interference from external factors: Spectroscopic imaging can be affected by external factors, such as temperature and humidity, which can impact the accuracy of the results (Table 2). Overall, spectroscopic imaging is a valuable tool for plant pathogen detection and diagnosis, but it has certain limitations that should be considered when using it for these purposes [139,140].

## 8. Detection and Diagnosis of *P. syringae* by AI (Artificial Intelligence) Methods

Machine learning is a type of artificial intelligence that uses image processing, algorithms, and statistical models to allow systems to learn from data without being explicitly programmed (Figure 3) [1,2,165]. There are several advantages to using machine learning techniques in plant pathogen detection and diagnosis. One advantage is that machine learning algorithms can analyze large amounts of data quickly and accurately, making it possible to analyze large datasets and identify patterns and trends that might not be easily discernible by humans. Additionally, machine-learning algorithms can be trained to recognize specific features or patterns in data, allowing them to accurately classify and predict outcomes in complex systems (Table 2). A study by Wang et al. (2022) used machine learning to analyze the genomic data from 101 *P. syringae* isolates and generated a predictive model for virulence affecting beans based on the information from 13 genes [144]. There are also some limitations to using machine learning in plant pathogen detection and diagnosis. One limitation is that machine-learning algorithms can only work with the data provided to them. Hence, the accuracy of their predictions is highly dependent on the quality and relevance of the data. Additionally, machine-learning algorithms may not be able to adapt to new or changing conditions, and they may require significant amounts of data and computing power to work effectively. Finally, there may be ethical concerns about using machine-learning algorithms to make decisions about plant health, as these decisions may significantly affect agriculture and food production (Table 2) [141,142,143,144,145]. Overall, machine-learning technology has the potential to significantly improve our ability to detect, diagnose, and control plant pathogens and can be a valuable tool for improving global food security [1,2,165]. Machine learning is thus far mainly used through image processing to detect and diagnose plant diseases. However, it has the potential to predict disease emergencies and development if previous years of disease development and epidemiology data are used for training the machine. A study by Li et al. (2022) used hyperspectral imaging and machine learning to classify healthy and diseased kiwi leaves infected by *Psa*, a strain of *P. syringae* that causes kiwifruit canker [166].

## 9. Koch’s Postulates as Golden Rules in the Detection and Diagnosis of *P. syringae*

**Figure 4:**
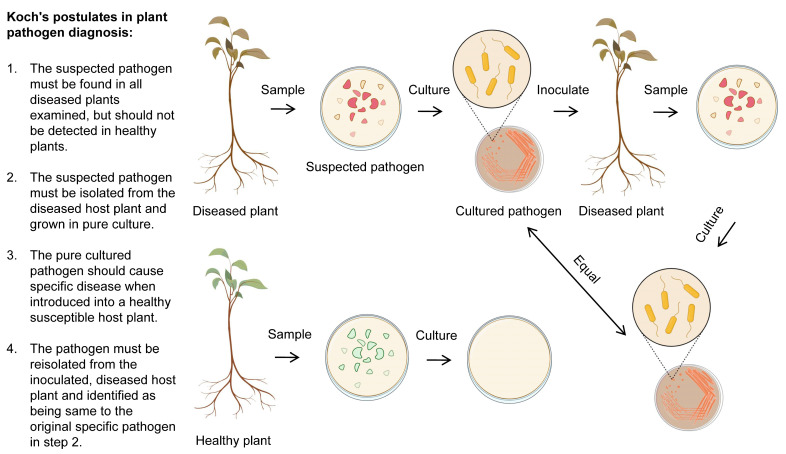
Koch’s postulates as golden rules in the detection and diagnosis of *P. syringae*. This figure was adapted from reference [168] with some modifications and additions. The figure was created with BioRender.com, accessed on 15 February 2023.

Koch’s postulates are a set of criteria used to prove the causal relationship between a specific microorganism and a particular disease. These postulates were developed by Robert Koch, a German bacteriologist, in the late 19th century, and they are still widely used in the detection and diagnosis of bacterial plant pathogens [167].The four postulates are as follows: (1) The microorganism must be present in every case of the disease. (2) The microorganism must be isolated from the infected plant and grown in pure culture. (3) The disease must be reproduced when the isolated microorganism is introduced into a healthy plant. (4) The same microorganism must be reisolated from the experimentally infected plant (Figure 4).

To apply Koch’s postulates in detecting and diagnosing a bacterial plant pathogen, a researcher would first find the presence of the pathogenic microorganism in a plant and then isolate the suspected causative microorganism from the infected plant tissue. The microorganism would then be grown in pure culture and used to infect a healthy plant, which should result in the development of the same disease symptom. Finally, the same microorganism should be isolated from the infected plant and confirmed to be consistent with the original isolate. If all four postulates are satisfied, it can be concluded that the microorganism is the causal agent of the disease [169].

While Koch’s postulates are widely used and considered a reliable method for establishing the causal relationship between a microorganism and a plant disease, they are not always applicable in all cases. For example, some microorganisms may be difficult to grow in pure culture, or the disease may not be reproducible in all cases. In these situations, alternative methods should be used to prove a causal relationship [170].

## 10. Conclusions

*P. syringae* is a bacterial plant pathogen that causes a wide range of diseases in very diverse plant species. These diseases, which can cause severe economic consequences, include bacterial speck, bacterial spot, bacterial canker, etc. Accurate and timely detection and diagnosis of *P. syringae* infections are critical for managing and controlling these diseases. Several methods can be used for the detection and diagnosis of *P. syringae* infections in plants. These methods can be broadly divided into traditional techniques and modern techniques. Traditional techniques for detecting and diagnosing *P. syringae* infections include culture isolation and microscopy approaches. Culture isolation involves the cultivation of *P. syringae* from plant tissues on specialized media. Microscopy involves the examination of plant tissues under a microscope to identify the presence of *P. syringae* cells. Modern techniques for the detection and diagnosis of *P. syringae* infections include PCR (polymerase chain reaction), ELISA (enzyme-linked immunosorbent assay), next-generation sequencing, etc. PCR is a molecular technique that can amplify and detect specific DNA sequences, including those of *P. syringae*. Serology involves the use of antibodies to detect the presence of *P. syringae* antigens in plant tissues. ELISA is a biochemical technique that can detect the presence of specific proteins, including *P. syringae* antigens, in plant tissues. Each method of detection and diagnosis has its advantages and disadvantages. Traditional techniques, such as culture isolation and microscopy are relatively simple and inexpensive. However, they can be time-consuming and may not be sensitive enough to detect low concentrations of *P. syringae*. Modern techniques, such as PCR and ELISA, have the advantage of being more sensitive and specific than traditional techniques. They can detect low concentrations of *P. syringae* and provide rapid results. However, they can be more expensive and require specialized equipment and training. In conclusion, different methods are available for the detection and diagnosis of *P. syringae* infections in plants. The choice of methods depends on the specific needs and resources of the laboratory or field setting. It is important to carefully consider the advantages and disadvantages of each method to select the most appropriate method for a given situation. Methods of detecting and diagnosing *P. syringae* will continue to evolve to meet the needs and challenges of the agricultural and greater scientific communities.

## 11. Future Directions on Preventive Management of *P. syringae*

**Figure 5:**
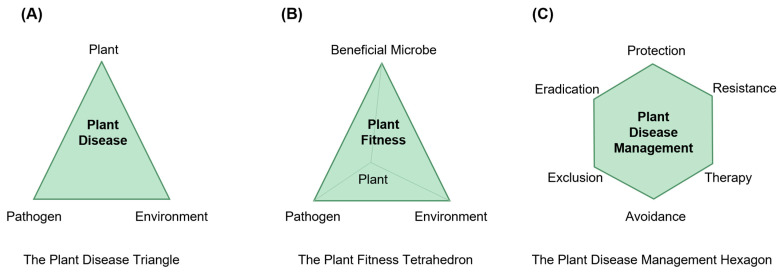
The conventional and current plant protection principles. (**A**) The conventional plant disease triangle model was adapted from reference [168]. (**B**) The current plant fitness tetrahedron model was adapted from references [171,172]. (**C**) The current plant disease management hexagon model was adapted from reference [173].

The conventional plant disease triangle is a model used to understand the complex interactions between a plant, a pathogen, and the environment that can lead to the development of a plant disease [168] (Figure 5A). The three components of the plant disease triangle are: (1) The host plant: This refers to the plant susceptible to the disease. The host plant has specific characteristics that make it more or less prone to the infection by a particular pathogen. (2) The pathogen: This refers to the microorganism that causes the disease. Pathogens can be bacteria, viruses, fungi, or other types of microorganisms. (3) The environment: This refers to the physical, chemical, and biological factors that promote the pathogenicity of pathogens. These factors include temperature, humidity, light, and nutrient availability [168] (Figure 5A).

The plant disease triangle model helps to illustrate that the development of plant disease results from the interactions between these three components. By understanding the relationships between these components, scientists and farmers can develop effective strategies for preventing and controlling plant diseases. However, the conventional plant disease triangle does not account for the potential contribution of beneficial microbes [168] (Figure 5A). Beneficial microbes are microorganisms that have a positive impact on the health and fitness of plants. Beneficial microbes can be found in the soil, on the surface of plants, and within plant tissues, including bacteria, fungi, viruses, and other microorganisms [174,175]. These microbes can promote plant growth and enhance plant defense by providing essential nutrients, protecting them from harmful pathogens, and increasing their resistance to abiotic stresses [9,176]. More and more studies have proved the significance and potential of beneficial microbes in promoting plant fitness [9,174,177,178,179]. Therefore, we propose a modified model called the plant fitness tetrahedron by integrating the beneficial microbes (Figure 5B). The presence of beneficial microbes is essential for the health and fitness of plants, and their importance has been increasingly recognized in agriculture and plant science [9,174,177,178,179]. There are several well-known ways in which beneficial microbes can enhance plant fitness. For example, some beneficial microbes can fix nitrogen, making it available to plants in a form they can use [180,181]. The other beneficial microbes can help plants absorb and use other essential nutrients, such as phosphorus and sulfur [182,183,184]. In addition, beneficial microbes can help plants defend themselves against harmful pathogens by competing for resources and secreting antimicrobial compounds [9,174]. Finally, beneficial microbes can help plants cope with environmental stressors, such as drought and extreme temperatures, by modulating their metabolism and signaling pathways; and protecting against plant’s oxidative damage [185,186,187,188].

Since the start of agriculture, farmers have continuously been improving their practices for combating various plagues suffered by crops [16,173,189,190]. Our growing understanding of the interactions between pathogens and hosts, which began with discovering the causes of plant diseases in the early 19th century, has allowed us to develop many methods for controlling specific plant diseases. Based on our more profound understanding of plant disease control, we have developed a set of general principles that can assist us in addressing new problems with any crop in certain environmental conditions [16,172,173,191]. Initially outlined by H. H. Whetzel and modified by various authors, these principles have been widely accepted and taught to plant pathology students, researchers, and other stakeholders globally [192]. In this review paper, we termed the core principles as the plant disease management hexagon (Figure 5C). The avoidance principle is to prevent plant diseases by selecting a time or location where the environment is not favorable for the pathogen infection or where there is no pathogenic inoculum. The exclusion principle is to prevent the introduction of the pathogenic inoculum. The eradication principle is to eliminate, inactivate, or destroy the pathogenic inoculum. The protection principle is to prevent infection through toxicants or other barriers to pathogenic infection. The resistance principle is to harness plant cultivars with inborn resistance or tolerance to pathogenic infections. The therapy principle is to cure plants already under the pathogenic infection [191]. The plant disease management hexagon is based on a comprehensive understanding of the interactions between the pathogen, the beneficial microbes, the host plant, and the environment. The effective management of plant diseases requires a combination of those principles tailored to the specific needs of each plant and the environment in which it is grown (Figure 5).

To effectively control *P. syringae* infections, in addition to the effective pathogen detections approaches, it is crucial to use disease-free seeds, practice crop rotation, and apply chemical or biological control agents as needed [34]. The plant fitness tetrahedron and the plant disease management hexagon (Figure 5) should also be considered to sustainably obtain effective controls.

## Figures and Tables

**Table 1:** Documentary records of plant diseases caused by *P. syringae*. Data were retrieved from NCPPB (National Collection of Plant Pathogenic Bacteria, https://www.fera.co.uk/ncppb, accessed on 15 February 2023).

Pathovar	Country	Host	Plant Symptoms	Year	References
pv. *aceris*	Japan	*Acer buergerianum Miq. ‘Tohkaeda’*(trident maple)	Necrotic leaf spots	1990	NCPPB, [35]
pv. *actinidiae*	Japan	*Actinidia chinensis* (kiwifruit)	Stem cankers	1990	NCPPB, [36]
pv. *aesculi*	India	*Aesculus indica* (horse chestnut)	Stem cankers	1990	NCPPB, [37]
pv. *antirrhini*	UK	*Antirrhinum majus* (snapdragon)	Necrotic leaf spots	1966	NCPPB, [38]
pv. *apii*	USA	*Apium graveolens var. dulce* (celery)	Necrotic leaf spots	1964	NCPPB, [39]
pv. *aptata*	USA	*Beta vulgaris* (sugar beet)	Tissue blights	1961	NCPPB, [40]
pv. *atrofaciens*	New Zealand	*Triticum aestivum* (bread wheat)	Glume rots	1974	NCPPB, [41]
pv. *atropurpurea*	Japan	*Lolium multiflorum* (ryegrass)	Shoot-tip diebacks	1971	NCPPB, [42]
pv. *avellanae*	Greece	*Corylus avellana* (hazel)	Stem cankers	1987	NCPPB, [43]
pv. *avii*	UK	*Prunus avium* (wild cherry)	Necrotic leaf spots	1959	NCPPB, [44]
pv. *berberidis*	New Zealand	*Berberis* sp. (barberry)	Necrotic leaf spots	1975	NCPPB, [45]
pv. *broussonetiae*	Japan	*Broussonetia kazinoki* (kozo)	Shoot blights	2008	NCPPB, [46]
pv. *cannabina*	Hungary	*Cannabis sativa* (hemp)	Leaf and stem rots	1960	NCPPB, [47]
pv. *castaneae*	Japan	*Castanea crenata* (chestnut)	Leaf blights	2008	NCPPB, [48]
pv. *cerasicola*	Japan	*Prunus × yedoensis (cherry tree)*	Galls on trunks and twigs.	2008	NCPPB, [49]
pv. *ciccaronei*	Italy	*Ceratonia siliqua* (carob)	Stem cankers	1971	NCPPB, [50]
pv. *coriandricola*	Germany	*Coriandrum sativum var. micocarpur* (coriander)	Necrotic leaf spots	1991	NCPPB, [51]
pv. *coronafaciens*	UK	*Avena sativa* (oat)	Leaf blights	1958	NCPPB, [52]
pv. *coryli*	Italy	*Corylus avellena* (hazel)	Stem cankers	2001	NCPPB, [53]
pv. *cunninghamiae*	China	*Cunninghamia lanceolata* (Chinese fir)	Small brown spots with yellow halos on needles (leaves)	2008	NCPPB, [54]
pv. *daphniphylli*	Japan	*Daphniphyllum teijsmanni* (himeyuzuriha)	Galls on trunks and twigs.	1989	NCPPB, [55]
pv. *delphinii*	New Zealand	*Delphinium* sp. (candle larspur)	Stem cankers	1966	NCPPB, [56]
pv. *dendropanacis*	Japan	*Dendropanax trifidus* (ivy)	Stem cankers	1986	NCPPB, [57]
pv. *dysoxyli*	New Zealand	*Dysoxylum* sp. (kohekohe)	Frost damages	1966	NCPPB, [58]
pv. *eriobotryae*	USA	*Eriobotrya japonica* (loquat)	Spots and blisters on fruit	1970	NCPPB, [59]
pv. *garcae*	Brazil	*Coffea arabica* (coffee)	Leaf and stem rots	1958	NCPPB, [60]
pv. *glycinea*	New Zealand	*Glycine max* (soybean)	Leaf blights	1971	NCPPB, [61]
pv. *helianthi*	Mexico	*Helianthus annuus* (sunflower)	Necrotic leaf spots	1974	NCPPB, [62]
pv. *hibisci*	USA	*Hibiscus rosa seinensis* (hibiscus)	Necrotic leaf spots	1990	NCPPB, [31]
pv. *japonica*	Japan	*Hordeum vulgare* (barley)	Leaf blights	1979	NCPPB, [63]
pv. *lachrymans*	Hungary	*Cucumis sativus* (cucumber)	Necrotic leaf spots	1960	NCPPB, [64]
pv. *maculicola*	New Zealand	*Brassica oleracea var. botrytis* (cauliflower)	Necrotic leaf spots	1967	NCPPB, [65]
pv. *mellea*	Japan	*Nicotiana tabacum* (tobacco)	Necrotic leaf spots	1971	NCPPB, [66]
pv. *mori*	Hungary	*Morus alba* (mulberry)	Necrotic leaf spots	1961	NCPPB, [67]
pv. *morsprunorum*	Switzerland	*Prunus armeniaca* (apricot)	Dead dormant buds	1971	NCPPB, [68]
pv. *myricae*	Japan	*Myrica rubra* (yumberry)	Necrotic leaf spots	1981	NCPPB, [69]
pv. *oryzae*	Japan	*Oryza sativa* (rice)	Sheath brown rots	1990	NCPPB, [70]
pv. *papulans*	Canada	*Malus sylvestris* (forest apple)	Blister spots	1975	NCPPB, [71]
pv. *passiflorae*	New Zealand	*Passiflora edulis* (passion fruit)	Necrotic leaf spots	1963	NCPPB, [72]
pv. *persicae*	France	*Prunus persica* (peach)	Stem cankers	1975	NCPPB, [73]
pv. *phaseolicola*	Canada	*Phaseolus vulgaris* (bean)	Necrotic leaf spots	1941	NCPPB, [74]
pv. *philadelphi*	UK	*Philadelphus coronarius* (dogwood)	Necrotic leaf spots	1983	NCPPB, [75]
pv. *photiniae*	Japan	*Photinia glabra* (Japanese photinia)	Necrotic leaf spots	1990	NCPPB, [76]
pv. *pisi*	New Zealand	*Pisum sativum* (pea)	Necrotic leaf spots	1974	NCPPB, [77]
pv. *porri*	France	*Allium porrum* (leek)	Leaf blights	1985	NCPPB, [78]
pv. *primulae*	USA	*Primula* sp. (primrose)	Necrotic leaf spots	1939	NCPPB, [31]
pv. *rhaphiolepidis*	Japan	*Raphiolepis umbellata* (yeddo hawthorne)	Necrotic leaf spots	1989	NCPPB, [79]
pv. *ribicola*	USA	*Ribes aureum* (golden currant)	Necrotic leaf spots	1961	NCPPB, [80]
pv. *nerii*	Spain	*Nerium oleander* (oleander)	Brown leaf galls	1983	NCPPB, [81]
pv. *sesami*	Greece	*Sesamum indicum* (sesame)	Necrotic leaf spots	1961	NCPPB, [82]
pv. *solidagae*	Japan	*Solidago altissima* (goldenrod)	Defoliation and terminal diebacks	2009	NCPPB, [83]
pv. *striafaciens*	USA	*Avena* sp. (oats)	Stripe blights	1966	NCPPB, [84]
pv. *syringae*	Japan	*Hordeum vulgare* (barley)	Leaf blights	1979	NCPPB, [85]
pv. *tabaci*	Australia	*Glycine max* (soybean)	Necrotic leaf spots	1975	NCPPB, [86]
pv. *tagetis*	Zimbabwe	*Tagetes erecta* (marigold)	Necrotic leaf spots	1972	NCPPB, [1]
pv. *theae*	Japan	*Thea sinensis* (tea plant)	Shoot blights	1974	NCPPB, [87]
pv. *tomato*	UK	*Lycopersicon esculentum* (tomato)	Necrotic leaf spots	1961	NCPPB, [32]
pv. *tremae*	Japan	*Trema orientalis* (charcoal-tree)	Necrotic leaf spots	1986	NCPPB, [88]
pv. *ulmi*	Yugoslavia	*Ulmus* sp. (elm)	Necrotic leaf spots	1959	NCPPB, [89]
pv. *viburni*	USA	*Viburnum* sp. (cranberry bush)	Leaf and stem spots	1966	NCPPB, [90]
pv. *zizaniae*	USA	*Zizania aquatica* (wild rice)	Leaf streaks	1990	NCPPB, [91]

**Table 2:** Comparison of methods for detection and diagnosis. This table was adapted from references [24,98,99] with some modifications and updates.

Method Type	Method	Advantages	Limitations	References
Conventional	Visual examination	Quick and easy to perform, onsite disease detection and diagnosis	Subjective, not sensitive enough at early stages	[96,97]
Conventional	Microscopy	High resolution, versatility	Sample preparation, sample size, shallow depth of field	[100,101,102]
Conventional	Culture plate	Relatively inexpensive, easy to use, isolation of individual bacterial species	Prone to contamination, not suitable for unculturable bacteria	[103,104]
Conventional	Phage typing	High specificity	Limited to certain bacteria, limited resolution, risk of contamination	[105,106,107]
Molecular	RPA (recombinase polymerase amplification)	High sensitivity, high specificity, rapid turnaround time, onsite disease detection and diagnosis	Limited multiplexing, low throughput, poor stability, high cost	[108,109,110]
Molecular	LAMP (loop-mediated isothermal amplification)	Onsite disease detection and diagnosis, simplicity	Limited multiplexing, limited commercial availability	[108,111]
Molecular	NGS (next-generation sequencing)	High throughput, large-scale, high resolution, versatility	Technical expertise, sample quality, data analysis, limited access	[112,113]
Molecular	FISH (fluorescence in-situ hybridization)	High sensitivity, high specificity, rapid, Easy to visualize	Photobleaching, autofluorescence, limited to specific sequences	[114,115]
Molecular	PCR (polymerase chain reaction)	Ease of use, quantitation possible, sensitivity, specificity, speed, versatility	PCR system affects the effectiveness, complexity, false positives	[104,116,117]
Serological	FCM (flow cytometry)	High throughput, multiple parameters, sensitivity, accuracy	Complexity, sample preparation, limited cell types, interference	[118,119,120]
Serological	ELISA (enzyme-linked immunosorbent assay)	Speed, ease of use, testing seed health, sensitivity, specificity, versatility	Expensive, complexity, limited scope, false positives	[121,122,123]
Serological	IF (immunofluorescence)	Sensitive and visualizable, multiplexing, ease of use	Photobleaching, requirement for specific antibodies, limited to surface-exposed molecules, need for specialized equipment	[102,124,125]
Serological	ImmunoStrip	Rapid, sensitive, specific, portable, easy to use	Specificity varies among products, expensive, a qualitative test,	[122,126]
Biomarker-based	Plant metabolite profiling	High specificity, early detection, high sensitivity, multiplexing capability, High throughput	Expensive, incomplete database, data analysis skills, complexity	[127,128,129]
Biomarker-based	Pathogen metabolite profiling	High specificity, early detection, high sensitivity, multiplexing capability, high throughput	Expensive, incomplete database, data analysis skills, limited to specific stages of infection, limited to specific pathogens	[130,131,132,133]
Biomarker-based	Microbiome analysis	High throughput, early detection, broad coverage	Expensive, incomplete database, data analysis skills, complexity, limited understanding, technical challenges	[128,134]
Vision-based	Hyperspectral imaging	Early-detection, can be used to study the effects of various factors, such as environmental conditions or treatment, on plant growth and development	Expensive, can be affected by factors such as light intensity and wavelength, may require the use of specialized equipment and software	[135,136,137,138]
Vision-based	Spectroscopic imaging	Early-detection, non-destructive, high spatial resolution, high sensitivity, rapid analysis	Expensive, limited depth penetration, complex sample preparation, need for calibration, interference from external factors	[139,140]
AI (artificial intelligence)	Machine learning	Speed, accuracy, cost-effectiveness	Lack of database, lack of algorithms, lack of understanding, dependence on high-quality data, lack of interpretability, bias	[141,142,143,144,145]

## Data Availability

Not applicable.
